# Models for financing the regulation of pharmaceutical promotion

**DOI:** 10.1186/1744-8603-8-24

**Published:** 2012-07-11

**Authors:** Joel Lexchin

**Affiliations:** 1 School of Health Policy and Management, York University, 4700 Keele St., Toronto, ON, M3J 1P3, Canada; 2 Emergency Physician, University Health Network, Toronto, Ontario, Canada; 3 Department of Family and Community Medicine, University of Toronto, 4700 Keele St., Toronto, ON, M3J 1P3, Canada

**Keywords:** Financing, Medications, Principles, Promotion, Regulation

## Abstract

Pharmaceutical companies spend huge sums promoting their products whereas regulation of promotional activities is typically underfinanced. Any option for financing the monitoring and regulation of promotion should adhere to three basic principles: stability, predictability and lack of (perverse) ties between the level of financing and performance. This paper explores the strengths and weaknesses of six different models. All these six models considered here have positive and negative features and none may necessarily be ideal in any particular country. Different countries may choose to utilize a combination of two or more of these models in order to raise sufficient revenue. Financing of regulation of drug promotion should more than pay for itself through the prevention of unnecessary drug costs and the avoidance of adverse health effects due to inappropriate prescribing. However, it involves an initial outlay of money that is currently not being spent and many national governments, in both rich and poor countries, are unwilling to incur extra costs.

## Background

Pharmaceutical companies spend vast sums promoting their products. In Italy in 1998 the figure was US $1.1 billion [1] and in the United States (US) companies spent US $57.5 billion in 2004 [2]. Promotion to doctors has been extensively studied and all forms – receiving information that originates with drug companies, using samples, taking gifts – is almost never associated with better prescribing [3-5]. The poor quality of medical journal advertising is a global issue [6]. Direct-to-consumer advertising (DTCA) is costly and drives up spending on medications; the 50 drugs that were the subject of DTCA accounted for almost as much of the increase in US spending on pharmaceuticals as the 10,000 that were not advertised directly to the public [7]. DTCA has never been reliably shown to improve compliance, lead to more appropriate early diagnosis of under-treated conditions, or prevent hospitalizations and serious disease consequences [8]. There is also indirect evidence that DTCA leads to negative health outcomes. Requests by “standardized” patients for a prescription for paroxetine (Paxil, a selective serotonin reuptake inhibitor antidepressant) for symptoms of adjustment disorder lead to a prescription in 55% of the cases despite the fact that medications are not indicated for the diagnosis [9]. Exposing patients to medications that will not benefit them means that no side effects are justified.

By law, companies are only allowed to promote drugs for uses for which the national regulatory authority has approved their use. Illegal off-label promotion has both negative health and economic consequences. Eli Lilly illegally promoted olanzapine (Zyprexa) for the treatment of dementia [10,11]. Nursing homes residents with dementia who are exposed to antipsychotic drugs such as olanzapine have a two times greater risk of an adverse event compared to those not taking these medications [12]. Illegal promotion of gabapentin (Neurontin) for off-label uses that had no scientific basis meant that sales rose from US $98 million in 1995 to nearly US $3 billion in 2004 [13]. Pfizer was fined $430 million in 2004 for its off-label promotion of gabapentin and its lawyers assured the US Attorney’s Office that the company would stop promoting drugs for unauthorized uses. Five years later Pfizer was once again fined, this time a then record of $2.3 billion for illegal promotion of valdecoxib (Bextra) and three other drugs [10]. The case of Pfizer illustrates that finding illegal promotion after it has occurred and then levying large fines is not enough to prevent the practice. Fines do not work because the profits to be made from this activity are much larger than the fines [10].

In countries such as the US where there is direct government control over promotion the number of people and the amount of money spent are grossly inadequate. In 2002 the FDA created a Review Group to oversee DTCA of prescription drugs but there were only seven people to monitor 10,000 pieces of promotion [14]. By 2008, the Review Group had expanded to eleven people but at the same time the amount of material it had to review had increased to over 20,000 items annually [15]. Overall, the FDA receives in the order of 75,000 to 80,000 individual pieces of promotional material per year [16] and as of 2008 had just 50 full-time staff and a budget of $9 million [17], an amount of money that is dwarfed by what the pharmaceutical industry spends on promotion. The FDA has acknowledged that it can’t review all submissions because of the volume that it receives [18].

Most countries have delegated the authority to industry to voluntarily control its own promotion. In the United Kingdom where this type of regulation is practiced the Prescription Medicines Code of Practice Authority has an annual budget of about £1,000,000 [19] but in most countries with voluntary industry regulation the amount spent is unknown. Voluntary self-regulation seems an attractive option because, lacking government-industry adversariness, it is a flexible and cost-effective option [20]. The problem is that industry will always be tempted to exploit the privilege of self-regulation by producing a socially sub-optimal level of compliance with regulatory goals. Trade associations vested with the authority to regulate drug promotion have almost uniformly made few systematic efforts to either monitor the advertising practices of their members or to enforce compliance. The mission of trade associations is primarily to increase sales and profit. From the business perspective, self-regulation is mostly concerned with the control of anti-competitive practices. Therefore, when industrial associations draw up their codes of practice they deliberately make them vague or do not cover certain features of promotion to allow companies a wide latitude. Previous work on the regulation of drug promotion has identified the deficiencies in protecting public health in the various regulatory systems currently used and concluded that any regulatory system needs to be independent of the pharmaceutical industry [20,21]. But to date there has not been any systematic discussion of how to fund an independent regulatory authority and without financing in place little is likely to happen.

The purpose of this paper is to fill this gap in the literature by exploring various models for generating the revenue needed. Here promotion is defined as any type of direct communication regarding prescription medications between pharmaceutical companies and either doctors or patients, including company controlled educational events, the giving of gifts and the provision of drug samples.

This paper comes out of a project initially conceived by Health Action International (HAI) [22], a consumer-based non-governmental organization, on how to incorporate the World Health Organization's *Ethical Criteria for Medicinal Drug Promotion* into national legislation. As part of the project HAI convened an expert international panel on promotion of which the author was a member. The basic principles that should inform any funding model for the regulation of promotion were discussed in an iterative manner by the expert panel both in person and through e mail until a consensus was reached. Similarly, the different types of funding models were identified by the panel and through a review of the literature.

## Discussion

### Criteria for determining how much money is required

Controlling the promotion of medicines is essential for public health purposes. Needless to say, any such control requires resources – time, energy, expertise and money. The amount required to regulate the promotion of medicines will depend on the number of personnel, their qualifications and the support (computers, travel expenses, training, etc.) required. The level of resources will depend on a variety of factors including:

· The scope of promotional activities to be monitored and how much promotion takes place;

· Whether monitoring will be active (initiated by the regulatory authority) or passive (using a complaints-based system);

· Whether promotional material will be pre-screened, i.e., examined for compliance with the regulatory code, before being disseminated;

· Whether or not the regulatory body functions independently and needs to be financially self-sufficient or is part of a larger regulatory authority and can draw resources from its parent body.

### Fundamental aspects of any financing model

Financing needs to be both stable and predictable. If the financing is not predictable then the regulatory body cannot plan future activities. If the amount of money is not stable then the agency would not be able to guarantee the maintenance of standards and might be forced to cut back on its activities.

A third principle that must be respected in order to preserve the agency’s independence is that the level of financing must not be tied to performance criteria set up for the regulatory body. (Performance standards should reflect public health objectives not financial requirements.) In the US, the Food and Drug Administration (FDA) has a statutory requirement to complete its review of 90% of new drug applications within specific periods of time. If the FDA fails to meet that obligation then renewal of legislation that allows it to collect user fees from industry may be endangered. Carpenter et al [23] concluded that for drugs approved in the immediate pre-deadline period the FDA does a less thorough job of reviewing drugs in order to avoid crossing the deadline and potentially jeopardizing its revenue.

Imposing standards on regulatory bodies in the name of “efficiency” usually benefits the industry being regulated more than it benefits public health. Revenue to the Canadian drug regulatory agency will suffer if reviews of new drug applications are not completed within the targeted time. If the actual performance in a given fiscal year is more than 10% off the target, penalties apply. Fees are then reduced for the next reporting year by a percentage equivalent to the performance not achieved, up to a maximum of 50%; so if reviews are 20% over the time allowed fees will drop by 20% [24].

Thus the three fundamental aspects of any model for financing the monitoring and regulation of promotion are: stability, predictability and lack of (perverse) ties between the level of financing and performance. The next section of this paper looks at various models in light of these three criteria and discusses other strengths and weaknesses the models have.

### Financial models

Six possible models are available to generate revenue for regulating promotion (Table 1):

1. A fee paid by pharmaceutical companies for each unique piece of promotional material (pamphlet, direct mail, advertisement, written or visual content of company-controlled continuing education, etc.) that they create or an overall tax on promotion;

2. A fee paid by pharmaceutical companies to regulatory authorities when drugs are submitted for approval and/or annual fees paid for drugs already on the market;

3. A fee from payers (government, insurance plans, individuals) or pharmaceutical companies for each prescription dispensed;

4. A fine paid by pharmaceutical companies for violations of the regulatory code;

5. Public funding from general tax revenue;

6. Payment from social insurance/mutual insurance funds.

**Table 1:** Comparison of financial models against the three fundamental criteria

**Approach**	**Criteria**			**Other features of approach**
	**Stability of funding**	**Predictability of funding**	**Preserves independence**	
Fee for each item of promotion	Yes, unless dramatic decrease in overall promotion or amount spent on promotion	Yes, unless dramatic decrease in overall promotion or amount spent on promotion	May create close relationship between industry and regulator	In Canada industry cooperation is voluntary
Fee paid by companies to regulatory authority to examine new drug applications and/or annual licensing fees	Not necessarily, would depend on the number of new drug applications submitted annually	Not necessarily, would depend on the number of new drug applications submitted annually	May create close relationship between industry and regulator	In the Australian variant some of the money is returned to the industry association to help fund its self-regulatory system
Fee for every prescription dispensed	Yes	Yes	Yes, but potentially not if companies pay a fee for every prescription dispensed for their products	Can be seen as a tax on the poor and sick; May not be feasible in developing countries where drugs are often bought through informal channels; Would not work in developed countries where the public health system covers most of the cost of medications
Fines paid by companies for code violations	Not necessarily, depends on how stringent code provisions are and how vigilant enforcement is	Not necessarily, depends on how stringent code provisions are and how vigilant enforcement is	Yes	Could encourage vigorous enforcement of code to increase revenue to regulator; Might be useful as a supplementary source of income
Using tax revenue	May depend on relationship between government and industry	May depend on relationship between government and industry	May depend on relationship between government and industry	Financing could be tied to health care system savings due to better regulation of promotion; Proper drug use might not necessarily lead to savings
Payments from social insurance funds	Depends on whether payments are made on voluntary basis or mandated by legislation	Depends on whether payments are made on voluntary basis or mandated by legislation	Yes	Financing could be tied to health care system savings due to better regulation of promotion

#### Fee for each piece of printed promotional material or an overall tax on promotion

This is the model used by the Pharmaceutical Advertising Advisory Board (PAAB) in Canada. Companies that belong to Canada’s Research-Based Pharmaceutical Companies (Rx&D) have voluntarily agreed to submit all printed material to the PAAB before it is circulated thus allowing material to be prescreened. The PAAB evaluates the material based on its Code of Advertising Acceptance [25] and either approves it for use or sends it back to the company for modification. There is a fee for each piece of promotional material and these fees cover all of the PAAB’s operating costs making it financially independent. Fees are adjusted depending on the resources required to run the organization.

Since the PAAB is dependent on the voluntary cooperation of the membership of Rx&D it is theoretically possible that the membership could decide to end its relationship with the PAAB but that is highly unlikely as it might trigger direct government intervention in the regulation of promotion. Therefore, the income that the PAAB receives is likely to be stable and predictable barring a major change in the amount of promotion undertaken by companies. If other countries chose to adopt this model they should use legislation to require that all material be submitted for prescreening before marketing rather than relying on voluntary cooperation. If there is a significant shift from printed promotion to other forms, e.g., company-sponsored continuing medical education, then the PAAB’s revenue could decline but so far that has not happened. Additionally, some countries may not have a sufficient volume of printed promotional material to make this a feasible way of financing a regulatory agency.

A major drawback to this form of revenue generation is the close relationship between the PAAB and the drug companies. While not identical to a system where income depends on performance, tying funding exclusively to fees from the pharmaceutical industry may create a situation where the regulator is too close to the companies that it regulates. In the case of the PAAB it may feel obligated to approve a certain percentage of material submitted to it or when it sees questionable material may be willing to give companies the benefit of the doubt. So far there is no empirical data to support these concerns.

Significant weaknesses in the PAAB code can lead to deceptive advertising that could compromise public health. These deficiencies may reflect either the fact that industry directly finances the PAAB, the composition of the PAAB board with its industry representation or a combination of both. Among others, examples of problems with the PAAB code are: detailed prescribing information does not have to appear with the display advertising; statements about intermediate outcomes are allowed as long as it is stated that their clinical significance is unknown.

Italy levies a 5% tax on the yearly promotional expenditures of all international and national pharmaceutical companies that target Italian health professionals. This money is then used to finance an independent program of research on drugs [26]. A similar tax could be used to fund the regulation of promotion.

#### Fee paid by pharmaceutical companies to the regulatory authority

Under a memorandum of understanding, the Medicines and Healthcare products Regulatory Authority (MHRA) (United Kingdom) through its Advertising Standards Unit (ASU) shares jurisdiction over the control of promotion for prescription drugs with the Prescription Medicines Code of Practice Authority, an industry affiliated body. The MHRA derives its total income from licensing fees paid by the pharmaceutical industry and uses some of the money to run the ASU.

In a variant of this system, a portion of the fees paid by pharmaceutical companies to the Australian Therapeutic Goods Administration are given back to the industry association, Medicines Australia (MA). MA then uses this income plus membership dues, fees for evaluating some promotional items and fines to fund a self-regulatory system. [Ken Harvey, personal communication]

If the number of new drug applications submitted each year or the yearly expenditures on promotion fluctuate significantly then the income of a regulatory authority could also vary substantially from year to year. Under these circumstances the resources available to regulate promotion might not always be adequate. Tying resources to regulate promotion to income from the pharmaceutical industry potentially creates the situation whereby the regulatory agency might alter its decisions in order to retain its funding. Under these circumstances it is possible that industry priorities may compete with public health interests.

#### A fee for every prescription dispensed

This model of generating revenue has not been used by any agency. It is the equivalent to a sales tax added onto the cost of a prescription. Alternatively a fee could be levied on pharmaceutical companies for every prescription filled for one of their products. The number of prescriptions dispensed has consistently increased on a year by year basis in developed countries and therefore income from this source should be stable and predictable. If the fee is coming from the purchaser of the prescription, there would be no direct relationship between the agency and the pharmaceutical industry. This would not be the case if the industry were paying the fee.

Whether or not this model would raise sufficient funds would depend on the fee. Too small a fee would mean inadequate funding while too large a fee might be resisted by payers. Additionally, if individuals were paying the fee then this way of raising money could be seen as a tax on the sick and the poor.

Finally, this model might be problematic in two situations. In some countries a large proportion of medications are purchased through informal networks, e.g., street drug sellers, small shops, etc. Even if these groups did collect the fee, the presence of unlicensed and untrained drug sellers is highly likely to lead to inappropriate drug use, defeating the main purpose of regulating promotion. Unmonitored sales of medications would also mean that it would not be possible to know how much money to collect from industry. In most Western European countries and other developed countries such as Australia the national health system covers most of the cost of medications with patients contributing only a relatively small copayment and/or deductible. Asking the health system for the extra money would only amount to one public body funding another. Adding a tax to copayments and/or deductibles would raise little revenue.

#### Fines paid by pharmaceutical companies

Relying solely on fines as a way of funding a regulatory agency is fraught with problems. If the agency is doing a good job and companies know that they are likely to be caught if they violate the code then little money might be generated. At the same time, the agency still needs to be vigilant to discourage non-compliance and therefore it still needs a stable source of revenue. Conversely, if the agency is doing a poor job then few violations would be detected and revenue would be insufficient. If the regulatory code were too weak then companies could engage in deceptive practices without violating the code and incurring any penalties. If there is a pyramid of sanctions [27] whereby initial code violations only incur minimal financial penalties then revenues might also not be adequate.

It would be in the interests of the agency to be aggressive about monitoring compliance as detecting more instances of code violations would increase income. However, since it is not possible to predict from year-to-year how much money will be raised through fines they are unreliable as a primary income source but could act as a supplementary source of revenue.

#### Using tax revenue

Financing a regulatory body out of public tax revenue may seem like the ideal model since the agency is protecting public health, but there are potential drawbacks. Unless the government is willing to commit funds over a multi-year period then funding might not be stable during times of economic stress. Furthermore, funding might vary depending on the political orientation of the governing party and its relationship with the pharmaceutical industry. In this sense, agency funding is indirectly tied to its relationship with the pharmaceutical industry. In the US, the FDA has the direct authority to regulate promotion but as we have seen, it has been given inadequate resources to do so.

Another consideration for public funding is tying the financing of the regulatory agency to the amount of money that it saves by leading to better prescribing and drug use. Australia uses savings to its Pharmaceutical Benefits Scheme to determine the amount of money that it gives to the National Prescribing Service. This model would have the advantage of making the regulatory body diligent about ensuring that promotion is accurate and objective. One drawback to this system is it requires a way of determining the extent of the savings and the amount may not be sufficient to cover all the activities of the regulatory body. A second drawback is that proper use of medications may not necessarily lead to savings.

#### Payments from social insurance/mutual insurance funds

Many countries use social insurance or mutual insurance funds to pay for the cost of prescription medicines. Insurance funds have a clear interest in promoting appropriate drug prescribing and use to help ensure that patients get the best possible care and also because appropriate drug therapy is cost-effective care. It is thus in the interest of insurance organizations that accurate and objective messages are delivered to consumers and health care providers. Therefore, they may be open to funding agencies that regulate promotion.

The involvement of insurance funds could either be legislated or it could be voluntary. Legislation would assure long-term financing but such a law may be difficult to enact depending on the structure of national funds. Voluntary payments would logistically be easier but then long-term financing is not assured. Furthermore, arriving at an appropriate level of payment may also be difficult. Payments would need to be lower than the savings derived from controlling promotion but calculating savings from appropriate promotion would not be easy.

Like public funding, this model of financing regulatory agencies also encourages regulators to be aggressive in controlling promotion in order to increase their income. If the funds were not satisfied that the regulators were adequately controlling promotion they might be reluctant to continue making payments.

This payment model makes the most sense if it is linked to broader post-market surveillance activities and/or information provision and activities to promote more appropriate prescribing that could lead to fewer unnecessary and needlessly expensive prescriptions.

### Determining the effectiveness of the financing model

The objective behind regulating promotion is to try and ensure that it does not have a negative effect on the way that physicians prescribe and patients use medications. Therefore, the evaluation of any of these proposed models depends on the degree to which they give the authorities the resources to successfully monitor and control promotion. Ideally, this would involve measuring patient health from the use of prescription medications, but as a proxy authorities could look at the extent to which promotion affects prescribing and use. One approach to doing so could be to expose people from both groups to currently circulating examples of promotion and then survey them about their knowledge regarding the medication. Of course, promotion works synergistically and one piece adds on to the next so looking at the effect of single promotional items may not be sufficient. Therefore, longer-term approaches might involve periodic monitoring of how appropriately doctors are prescribing and patients are using medications that are the subject of on going promotion.

## Summary

All the six models considered here have positive and negative features and none may necessarily be ideal in any particular country. Different countries may choose to combine two or more of these models depending on their unique environments and laws. Moreover, a decision about which model or models to use should be seen as dynamic not static. As conditions change or if one model proves unsuccessful countries can adapt or abandon the model that they have chosen.

Financing of regulation of drug promotion should more than pay for itself by preventing extra unnecessary drug costs and avoiding adverse health effects caused by inappropriate prescribing. However, it involves an initial outlay of money that is currently not being spent and many national governments, both from rich and poor countries, are unwilling to incur extra costs. The recognition that it costs money to regulate promotion underscores the notion that regulatory systems, however financed, will only be possible if there is the political will and support from the public and health professionals to effectively control promotion.

Finally, whatever model is chosen it is important to retain the three fundamental principles: stability, predictability and ensuring that the funding source does not compromise performance.

## Competing interests

In 2007 Joel Lexchin was a consultant to a law firm acting for Apotex Inc. In 2008 he was an expert witness for the Canadian federal government in its defense against a lawsuit challenging the ban on direct-to-consumer advertising. In 2010 he was an expert witness for a law firm representing the family of a plaintiff who allegedly died from an adverse reaction from a product made by Allergan. He is currently on the Management Board of Healthy Skepticism Inc. and is the Chair of the Health Action International – Europe Association Board.

## Authors’ contributions

JL conceived of the idea for this paper, gathered all of the information, wrote and revised the manuscript.
